# Post-radiation sinusitis is associated with recurrence in nasopharyngeal carcinoma patients treated with intensity-modulated radiation therapy

**DOI:** 10.1186/s13014-019-1261-9

**Published:** 2019-04-11

**Authors:** Chih-Jen Huang, Ming-Yii Huang, Ming-Chen Paul Shih, Kai-yuan Cheng, Ka-Wo Lee, Tzu-Ying Lu, Shyng-Shiou Yuan, Pen-Tzu Fang

**Affiliations:** 1 Department of Radiation Oncology, Kaohsiung Medical University Hospital, No.100, Tzyou 1st Road, Kaohsiung, 807 Taiwan; 2 Department of Radiation Oncology, Faculty of Medicine, Kaohsiung Medical University, Kaohsiung, Taiwan; 3 Department of Medical imaging, Kaohsiung Medical University Hospital, Kaohsiung, Taiwan; 4 Department of Radiology, Faculty of Medicine, Kaohsiung Medical University, Kaohsiung, Taiwan; 5 Department of Otolaryngology-Head and Neck Surgery, Kaohsiung Medical University Hospital, Kaohsiung, Taiwan; 6 Translational Research Center, Department of Obstetrics and Gynecology, Kaohsiung Medical University Hospital, Kaohsiung, Taiwan

**Keywords:** Nasopharyngeal carcinoma, Intensity-modulated radiation therapy, Post-radiation sinusitis, Recurrence

## Abstract

**Background:**

This study investigated the impact of post-radiation sinusitis on the prognosis of nasopharyngeal carcinoma (NPC) patients treated with intensity-modulated radiation therapy (IMRT).

**Methods:**

Two hundred and thirty patients with non-metastatic NPC were analyzed in terms of freedom from local failure (FFLF), freedom from distant failure (FFDF), overall survival (OS), and disease-free survival (DFS). For each patient, the status of the sinus mucosa was flexibly assessed by documenting mucosal changes as indicated by differences between images obtained before radiotherapy and more than 6 months post-radiation.

**Results:**

With a median follow-up of 39.7 months (8 to 81 months), 19 (8.26%) patients relapsed locally, 13 (5.65%) patients failed in the neck, and 26 (11.3%) patients developed distant metastases. The presence of sinusitis noted in images post-radiation was a significant predictor for DFS (*p* = 0.001), FFLF (*p* = 0.004), and FFDF (*p* = 0.015), in addition to having high negative predictive value for local relapse (97.5%).

**Conclusions:**

This is the first study to investigate the prognostic value of post-radiation sinusitis in NPC patients treated with IMRT. Post-radiation sinusitis was found to be a significant predictor for DFS, FFLF, and FFDF, and was also found to have high negative predictive value for local recurrence (97.5%). It may thus be used as an additional tool for clinicians to determine the possibility of recurrence.

## Background

Nasopharyngeal carcinoma (NPC) is a common cancer in Taiwan and Southeast Asia. For early stage NPC, the standard treatment is radiotherapy (RT) alone. For the advanced stage of the disease, a combination of chemotherapy and RT is necessary [1]. Due to advances in RT techniques, improvements in treatment outcomes have been achieved. In the past decade, intensity-modulated radiotherapy (IMRT) has been proven to be a more effective means of treating NPC than conventional RT [2, 3]. This breakthrough technique allows dose escalation to the tumor and delivers a highly conformal dose distribution in treating NPC. Therapeutic gains have been achieved by simultaneously improving local control and reducing RT-related toxicity.

Clinicians are making ongoing efforts to discover useful prognostic factors in this new IMRT era, given that IMRT has already been shown to have different dosimetric characteristics than three-dimensional conformal radiation therapy (3D-CRT). Some researchers have analyzed the patterns of local and regional failure [4–6], while others have sought to identify different predictors for local recurrence [7–10]. For local recurrence, the T stage is still the most well recognized prognostic factor, while tumor volume is a somewhat controversial predictor of local recurrence and cutoff volume is still being investigated with regard to its predictive value [8, 9, 11, 12]. Most authors agree that distant metastasis continues to pose the most difficult treatment challenge despite the use of combined chemotherapy. After treatment, Epstein-Barr virus (EBV) DNA is helpful for surveillance [13–15]; however, the quantitative methods used in each laboratory are different, and the interpretation criteria vary for every facility. So, practitioners are making efforts to investigate new available and useful prognostic factors for the purposes of surveillance after treatment.

This study sought to investigate the prognostic factors of NPC patients treated with IMRT and was approved by the institutional review board at Kaohsiung Medical University Hospital.

## Methods

### Patient characteristics

Between November 2007 and June 2013, 230 histologically diagnosed non-metastatic NPC patients were treated with IMRT at Kaohsiung Medical University Hospital in Kaohisung, Taiwan. For each patient, various pretreatment evaluations were conducted, including a history and physical examination, dental evaluation, blood test, nasoendoscopy, computed tomography (CT) or magnetic resonance imaging (MRI) of the head and neck, chest X-ray, bone scan, and abdominal sonography or positron emission tomography (PET). Tumors were staged according to the 2010 American Joint Committee on Cancer (AJCC) staging classifications [16]. Histologic classifications were made according to the 2005 World Health Organization (WHO) pathologic classification [17].

### Radiotherapy

IMRT was delivered via either helical tomotherapy (HT; Accuray Incorporated, Sunnyvale, CA) or volumetric modulated arc therapy (VMAT; RapidArc, Varian Medical Systems, Palo Alto, CA). Patients were immobilized in a supine position with a thermoplastic mask covering the head and neck. A CT simulation with a slice thickness of 3 mm was performed, and target and normal structures were delineated on a Pinnacle treatment planning system (Phillips Healthcare, The Netherlands). The gross tumor volume (GTV) included the macroscopic primary tumor and involved lymph nodes of more than 10 mm in diameter. The clinical target volume high (CTVhigh) included the GTV with an expansion of 3 mm. The CTVmid was designed to include areas at risk for microscopic involvement, including the entire nasopharynx, the retropharyngeal nodal regions, the skull base, the clivus, the pterygoid fossa, the parapharyngeal space, the sphenoid sinus, the posterior third of the nasal cavity/maxillary sinuses and the pterygopalatine fossa, and lymph nodes at levels II–III, level VA, and the retropharyngeal space (ipsilateral level IB was included if the N stage was positive). Level IV and level VB were included in the CTVlow. The planning target volume (PTV) was defined as the CTV with 3-mm margins in all dimensions. However, in areas in which the CTV was adjacent to critical normal structures (e.g., the brainstem), the margin was reduced to 1 mm. The prescribed doses for PTVhigh, PTVmid, and PTVlow were 69.96-70Gy in 33–35 fractions, 59.4-63Gy in 33–35 fractions, and 54.45-56Gy in 33–35 fractions, respectively. The organs at risk (OAR) (i.e., the brainstem, spinal cord, lenses, eyes, optic nerves, chiasm, mandible, parotid glands, oral cavity, and throat) were contoured, and dose limitations were set modified based on the radiation therapy oncology group (RTOG) 0225 trial [18]. During treatment, kilovoltage cone beam CT (CBCT) image guidance for VMAT or megavoltage CT (MVCT) image guidance for HT was utilized to verify the tumor position.

### Chemotherapy

For stage II-IVB patients, combined chemotherapy was needed and was provided based on the clinician’s assessment, with the factors considered including the patient’s age, The Eastern Cooperative Oncology Group (ECOG) scale of performance status, co-morbidities, tumor extent, and the patient’s own preferences. Neoadjuvant chemotherapy (NACT) followed by RT, concurrent chemo-RT (CCRT), and neoadjuvant chemotherapy followed by concurrent chemo-RT (NACCRT) were all accepted treatment options. For patients receiving NACT, the chemotherapy was administered as 2–3 cycles of cisplatin 70 mg/m^2^ on day 1 and 5-fluorouracil 500–1000 mg/m^2^ on days 2–5, every 3 weeks. For patients receiving CCRT, 2–3 cycles of cisplatin 70 mg/m^2^ were delivered at 3-week intervals, or 6–7 cycles of cisplatin 30 mg/m^2^ were delivered weekly. Two patients received concurrent cetuximab with radiotherapy.

### Follow-up

After completion of the treatment, routine follow-ups were conducted every 3 months during the first 3 years, and then every 6 months thereafter. These follow-up evaluations consisted of a physical examination, nasoendoscopy, CT or MRI scan, chest x-ray, and abdominal sonography. Late toxicities were recorded in medical documents during follow up.Sinusitis was defined radiologically via CT scan or MRI scan [19–21]. The diagnosis criteria were as follows: enhanced scan showing fluid accumulation or opacification in the paranasal sinuses, or thickened sinus mucosa with trapped secretion, effusion, or air/fluid in the sinus cavity. Pre-radiation sinusitis was defined according to the imaging performed at the time of diagnosis, while post-radiation sinusitis was defined according to the imaging performed more than 6 months after the radiotherapy. Massive improvement from pre-radiation sinusitis was also regarded as negative post-radiation sinusitis. The occurrence of sinusitis was determined by CJH, MCPS and PTF.

### Statistical analysis

The statistical analysis was performed using SPSS 19.0 software. Several endpoints were evaluated: overall survival (OS), disease-free survival (DFS), freedom from local failure (FFLF), and freedom from distant failure (FFDF). The determinations of local relapse and distant metastasis were made based on physical examinations or radiographic images or were proven by pathological reports. The durations of DFS, FFLF, and FFDF were calculated from the date of completion of the main treatment to the date of documented failure, death from any cause, or the date of the last follow-up. The duration of OS was measured from the date of the diagnosis until death or the date of the last visit. The Kaplan-Meier method was used to calculate the cumulative OS, DFS, FFLF, and FFDF. Different prognostic factors were analyzed using the log-rank test. Among the statistically significant factors identified by univariate analysis, strongly related factors with *P* < 0.01 were selected for multivariate analysis. The Cox proportional-hazards model was used for multivariate analysis. The ENTER method was used. *P* < 0.05 was considered significant.

## Results

**Fig. 1:**
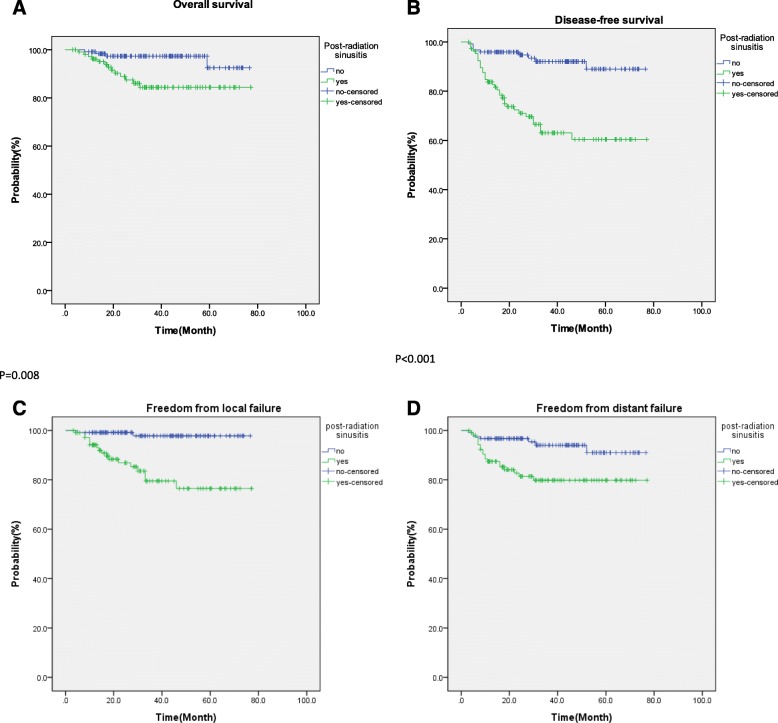
Analysis of the association between post-radiation sinusitis and **a**: overall survival (OS), **b**: disease-free survival (DFS), **c**: freedom from local failure (FFLF), and **d**: freedom from distant failure (FFDF)

**Fig. 2:**
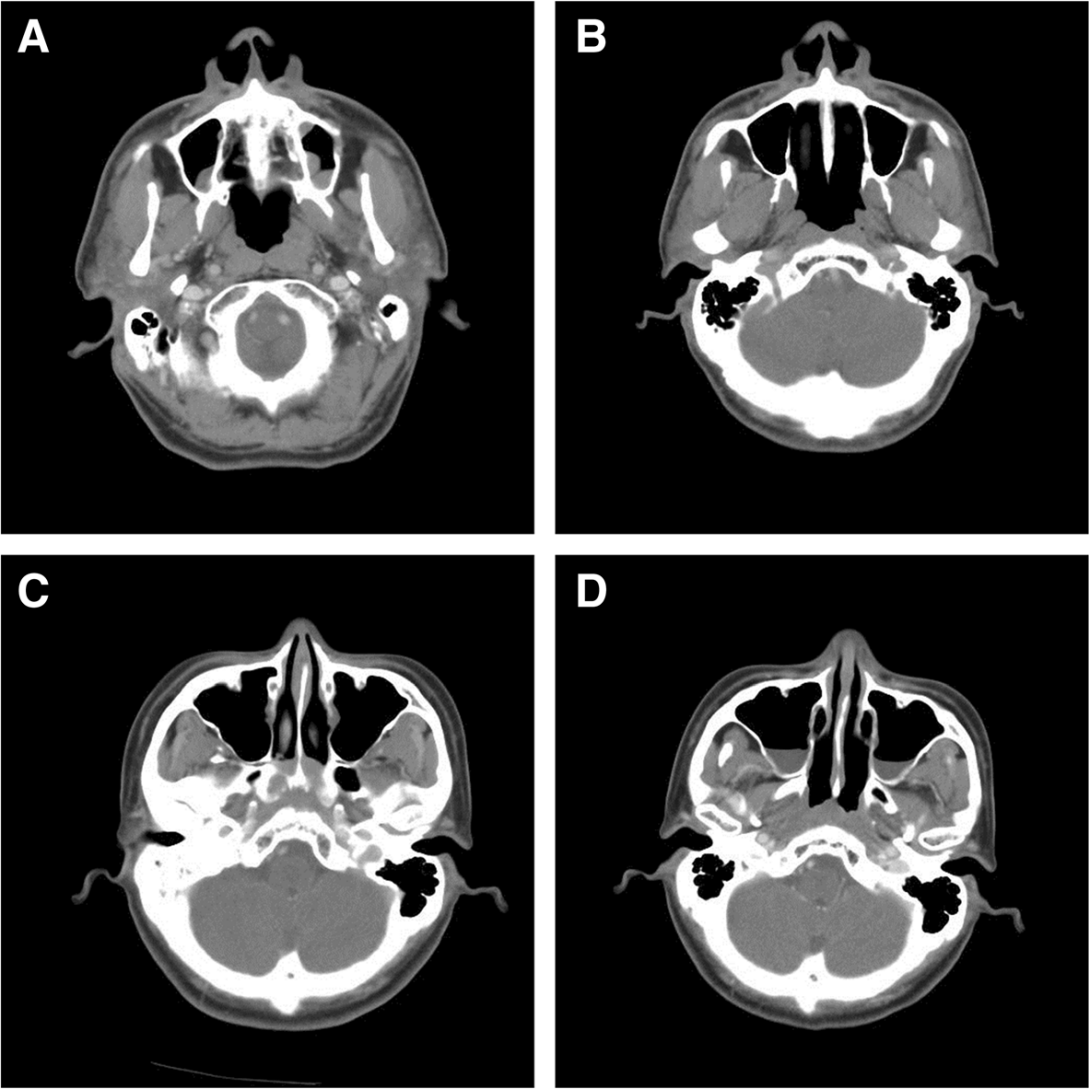
Changes of sinusitis as depicted by the differences between pre-RT and post-RT CT images. **a** is the pre-RT CT image of a patient who presented with bilateral maxillary sinusitis. **b** is a follow-up CT image of the same patient taken at 1 year post-RT. As shown, the bilateral sinusitis was diminished. This patient has been disease free for 5 years. **c** is the pre-RT CT image of another patient showing no evidence of sinusitis. **d** is a follow-up CT image of the same patient taken at 1 year post-RT. As shown, the patient had developed bilateral maxillary sinusitis at 1 year post-RT, and a biopsy confirmed local recurrence

A total of 230 patients with non-metastasis NPC were treated with IMRT at Kaohsiung Medical University Hospital between November 2007 and June 2013. The median age of these patients was 48.5 years (range: 18–80 years). One hundred seventy-seven of the patients were male, and 53 of the patients were female. Eighty-seven percent of the patients were in a locally advanced stage (stages II-IVB). The patient and disease characteristics are summarized in Table 1. With a median follow-up of 39.7 months (range: 8.2–81 months), the 3-year OS, DFS, FFLF, and FFDF rates were 91.4, 80.3, 90.6, and 87.5%, respectively. Nineteen local failures, 26 distant failures, and 17 deaths were observed, while a total of 43 relapses were noted. Among patients who experienced local recurrences and distant metastases, 63% developed local failure within 2 years, and 76.9% developed distant metastasis within 2 years. For the patients with post-radiation sinusitis versus those without post-radiation sinusitus, the 3-year OS, DFS, FFLF, and FFDF rates were, respectively, 97.4% versus 84.4%(*p* = 0.008), 92.1% versus 66.5% (*p* < 0.001), 97.9% versus 81.7% (p < 0.001), and 94.1% versus 79.8%(p < 0.001) (Fig. 1). Figure 2 shows the changes of sinusitis for these two groups of patients, i.e., those with and without post-radiation sinusitis. Before radiotherapy, the incidence of sinusitis among all the patients was 54.3%. After treatment, the incidence was 47%. Among the 125 patients with pre-radiation sinusitis, the sinusitis of 34 (27.2%) was alleviated after radiotherapy. Among the 105 patients without pre-radiation sinusitis, however, 17 (16.2%) developed post-radiation sinusitis. Furthermore, among the 122 patients who did not have post-radiation sinusitis, only 3 (2.5%) presented with local recurrence, meaning that the negative predictive value of post-radiation sinusitis for local recurrence was 97.5%.

**Table 1:** Patient and disease characteristics

Patient and Disease Characteristics	No (%)
Age Median (range)	48.5 (18–80)
Gender	
Male	177 (77)
Female	53 (23)
Histology	
WHO type 1	3 (1.3)
WHO type 2.1 undifferentiated	77 (33.6)
WHO type 2.2 differentiated	112 (48.7)
WHO type 2 NOS	37 (16.1)
WHO type 3 basaloid	1 (0.4)
AJCC 7th Stage	
I/II/III/IV	30 (13)/77 (33.5)/78 (33.9)/45 (19.6)
T stage	
1/2/3/4	106 (46.1)/61 (26.5)/34 (14.8)/29 (12.6)
N stage	
0/1/2/3	59 (25.7)/86 (37.4)/66 (28.4)/19 (8.3)
RT modality	
HT	155 (67.4)
VMAT	75 (32.6)
Treatment factors	
RT alone	38 (16.5)
NACT+RT/NACCRT	169 (73.5)
CCRT	23 (10)
Sinusitis	
Pre-radiation sinusitis	
No	105 (45.7)
Yes	125 (54.3)
Post-radiation sinusitis	
No	123 (53)
Yes	107 (47)

*Abbreviations*: *VMAT* volumetric modulated arc therapy, *HT* helical tomotherapy, *NACT* neoadjuvant chemotherapy, *NACCRT* neoadjuvant chemotherapy followed by concurrent chemoradiotherapy, *CCRT* concurrent chemoradiotherapy

The impact of various prognostic factors on the clinical outcomes was examined by univariate analysis. Various prognostic factors were evaluated, including age, gender, T stage, N stage, the use of chemotherapy, and sinusitis status. Univariate analysis using the log-rank test revealed that age > 40 years, male gender, T4 stage, N3 stage, and post-radiation sinusitis were significantly associated with poorer outcomes in terms of OS and DFS. T4 stage and post-radiation sinusitis, however, were the only prognostic factors associated with poor FFLF. For FFDF, meanwhile, male gender, T4 stage, N3 stage, and post-radiation sinusitis were the significant prognostic factors (Table 2).

**Table 2:** Univariate analysis of prognostic factors

	3 yr. OS (%)	p	3 yr. DFS (%)	p	3 yr. FFLF (%)	p	3 yr. FFDF (%)	p
Gender		0.025		0.009		0.06		0.018
Male	89		76.3		89		84.9	
Female	100		94.4		96.3		96.8	
Age		0.049		0.049		0.108		0.18
< 40y/o	88.9		89.9		96.3		93.7	
> 40y/o	88.4		76.7		88.5		85.2	
T classification		0.002		< 0.001		< 0.001		0.012
T1–3	94		84		93.8		89.4	
T4	72.2		54.1		66.5		73.7	
N classification		0.001		< 0.001		0.654		< 0.001
N0–2	93.1		82.8		89.8		90.2	
N3	71.6		51.7		88.9		57.4	
Chemotherapy		0.083		0.036		0.543		0.085
No	100		88.9		88.9		94.4	
Yes	90		78.5		90.7		86.1	
Pre-radiation sinusitis		0.048		0.002		0.057		0.082
No	96.4		89.4		93.7		91.4	
Yes	87.1		72.5		87.9		84.2	
Post-radiation sinusitis		0.008		< 0.001		< 0.001		0.003
No	97.4		92.1		97.9		94.1	
Yes	84.4		66.5		81.7		79.8	

*Abbreviations*: *p p*-value, *OS* overall survival rate, *DFS* disease-free survival rate, *FFLF* freedom from local failure, *FFDF* freedom from distant failure

Multivariate analysis using the Cox proportional-hazards model showed that N3 stage and T3 stage were significant prognostic factors for OS, while male gender, T4 stage, N3 stage, and post-radiation sinusitis were independent factors predicting recurrence. Furthermore, N3 stage and post-radiation sinusitis were shown to be independent factors predicting distant metastasis, while post-radiation sinusitis and T stage were the independent factors predicting local recurrence. Table 3 depicts the significant prognostic factors of different end points according to the multivariate analysis.

**Table 3:** Significant prognostic factors according to multivariate analysis

End point	Hazard ratio (95%CI)	p-value
Overall survival		
T stage T4 vs. T1–3	3.914 (1.441–10.63)	0.007
N stage N3 vs. 0–2	4.735 (1.655–13.547)	0.004
Disease-free survival		
Gender Male vs. Female	4.537(1.392–14.792)	0.012
T stage T4 vs. T1–3	2.705(1.359–5.384)	0.005
N stage N3 vs. 0–2	3.425 (1.671–7.019)	0.001
Post-radiation sinusitis Yes vs. no	3.734(1.732–8.051)	0.001
Freedom from local failure		
T stage T4 vs. T1–3	2.786(1.069–7.259)	0.036
Post-radiation sinusitis Yes vs. no	8.9441(2.002–39.965)	0.004
Freedom from distant failure		
N stage N3 vs. 0–2	4.982 (2.149–11.552)	< 0.001
Post-radiation sinusitis Yes vs. no	2.951 (1.233–7.064)	0.015

## Discussion

To our knowledge, this is the first study to have demonstrated a relationship between post-radiation sinusitis and local recurrence. Post-radiation sinusitis is not uncommon after RT. In past studies, the incidence of post-radiation sinusitis was found to be highest at 3 to 6 months after RT, ranging from roughly 50 to 75% in NPC survivors following RT, and then gradually decreasing thereafter [21–23]. In those studies, sinusitis referred to radiological evidence of sinus mucosal change as opposed to any aspect of the clinical presentation. In the present study, the presence of post-radiation sinusitis more than 6 months after treatment was one of the prognostic factors indicating poor outcomes in terms of DFS, FFLF, and FFDF. Because post-radiation sinusitis tends to decrease and stabilize between six months and about a year after treatment, we documented the presence or absence of sinusitis during this period. Moreover, it was also reasonable to use post-radiation sinusitis at 6 months to 1 year after radiotherapy as a predictor given that most cases of recurrence occurred within 2 years in the present study. Thus, the presence of sinusitis documented 6 months after RT may be good cutoff time point for clinical practitioners seeking to predict local recurrence. Figure 2 shows changes in the sinuses from before to after RT in two patients. The images in Fig. 2a and b show a patient whose sinusitis was diminished post-RT and who then remained disease-free for 5 years. In contrast, the images in Fig. 2c and d depict another patient who developed bilateral maxillary sinusitis by 1 year post-RT and then experienced local recurrence as confirmed by biopsy.

The etiology of post-radiation sinusitis is assumed to be epithelial cell degeneration and ciliary dysfunction. Increased secretions and suppressed excretion function lead to the retention of secretions. Structural changes such as choanal atresia, hypertrophy of the turbinates, and nasal adhesion also worsen the condition [21, 22]. IMRT does not increase the incidence and severity of post-radiation sinusitis when compared to conventional 3D CRT [24]. With regard to the correlation between radiation field and post-radiation sinusitis, expert researchers have expressed conflicting opinions [21, 24].

There are two possible reasons that can explain the relationship between post-radiation sinusitis and local recurrence. One possible explanation is an association between the inflammatory process and carcinogenesis owing to high EBV infection prevalence rate among Chinese people. A retrospective cohort study based on the National Health Insurance database of Taiwan noted that those patients who presented with rhinosinusitis were found to have a 3.55-fold increased risk of developing NPC compared with individuals without rhinosinusitis [25]. The author of that study suggested that sinonasal EBV infections cause precancerization in NPC patients with certain genetic variations and that these infections then present as chronic rhinosinusitis. Moreover, such preexisting sinusitis will not worsen after RT, but rather will decrease [24]. Further studies of EBV DNA from biopsied sinus mucosal tissue may give us more clues in support of this theory.Another possible reason is the differing susceptibilities of the bony and mucosal structures of the paranasal sinuses to tumors or to radiation. In general, those patients who did not present with post-radiation sinusitis had a robust osteomeatal complex and a healthier environment, and were less likely to suffer from local tumor relapse. Meanwhile, better innate immunity and less inflammation among those who did not present with post-radiation sinusitis may have caused those patients to be less susceptible to tumor recurrence. That being said, a better understanding of the mechanisms involved in immunity and the inflammation process is needed to confirm or disprove these theories.

The correlation between T stage and the severity of post-radiation sinusitis has been discussed previously [21]. However, unlike other studies which investigated severity using Lund scores, in the present study, we focused only on the “presence” or “absence” of sinusitis, and found that the correlation between the presence of post-radiation sinusitis and T stage was weak (r = 0.303). We can also note that, according to the multivariate analysis, T stage and post-radiation sinusitis were both statistically significant in terms of their associations with DFS and FFLF (Table 3), so the interference of T stage is weak, meaning that using the presence or absence of post-radiation sinusitis as a prognostic indicator should be simple for clinicians.

We noted that DFS and FFDF were also significantly associated with post-radiation sinusitis; however, we have no clear explanation for these observed associations. That said, it is reasonable to speculate that post-radiation sinusitis is related to depressed innate immunity and elevated inflammatory status, which may contribute in turn to augmented micro-metastasis and eventually lead to a generalized deterioration in disease control and distant metastasis.

There is no doubt that IMRT is currently the treatment of choice for NPC, because it can provide superior dose conformity to the target and better protection of surrounding normal organs than 2D RT [18, 26]. These dosimetric advantages of IMRT can be translated into better clinical outcomes. Some authors have even advocated that IMRT can partially fill the role of chemotherapy [15]. However, in advanced stage NPC, combined chemotherapy is still the standard form of treatment. It is worth mentioning that, in the present study, most of the stage II-IVB patients (73.5%) received NACT followed by RT or NACCRT, indicating that NACT showed promising results, at least in the endemic area [27–29]. Furthermore, the OS, DFS, FFLF, and FFDF of the patients in the present study were comparable to those of patients in previous studies [2, 26, 30–34].

This study had limitations. First, it was a retrospective study based solely on clinical observations. Second, by simply documenting the presence or absence of sinusitis via image studies 6 months after radiotherapy, it presents a straightforward tool for clinicians; however, future studies should use a more rigorous definition of sinusitis, a more sophisticated analysis of the exact cutoff timing of sinusitis, and/or correlate the sinusitis with severity using the Lund-Mackay system. Third, with regard to the high negative predictive value, we can only be sure that the likelihood of local recurrence was low for those who patients who had no post-radiation sinusitis. In contrast, for those who presented with post-radiation sinusitis, the positive predictive value was still low.

## Conclusion

This study demonstrated that post-radiation sinusitis is one of the prognostic factors for poor DFS, FFLF, and FFDF, and that it has high negative predictive value for local recurrence. The reasons for this may be differing levels of patient immunity, the inflammatory process related to EBV infection, and structural abnormalities. This study still requires further validation.
